# Sugarcane juice derived carbon dot–graphitic carbon nitride composites for bisphenol A degradation under sunlight irradiation

**DOI:** 10.3762/bjnano.9.35

**Published:** 2018-01-30

**Authors:** Lan Ching Sim, Jing Lin Wong, Chen Hong Hak, Jun Yan Tai, Kah Hon Leong, Pichiah Saravanan

**Affiliations:** 1 Department of Environmental Engineering, Faculty of Engineering and Green Technology, Universiti Tunku Abdul Rahman, Jalan Universiti, Bandar Barat, 31900, Kampar, Perak, Malaysia; 2 Environmental Nanotechnology Laboratory, Department of Environmental Science and Engineering, Indian Institute of Technology (ISM), Dhanbad 826004, Jharkhand, India

**Keywords:** carbon dots, g-C_3_N_4_, photocatalytic degradation, sugarcane juice, sunlight

## Abstract

Carbon dots (CDs) and graphitic carbon nitride (g-C_3_N_4_) composites (CD/g-C_3_N_4_) were successfully synthesized by a hydrothermal method using urea and sugarcane juice as starting materials. The chemical composition, morphological structure and optical properties of the composites and CDs were characterized using various spectroscopic techniques as well as transmission electron microscopy. X-ray photoelectron spectroscopy (XPS) results revealed new signals for carbonyl and carboxyl groups originating from the CDs in CD/g-C_3_N_4_ composites while X-ray diffraction (XRD) results showed distortion of the host matrix after incorporating CDs into g-C_3_N_4_. Both analyses signified the interaction between g-C_3_N_4_ and CDs. The photoluminescence (PL) analysis indicated that the presence of too many CDs will create trap states at the CD/g-C_3_N_4_ interface, decelerating the electron (e^−^) transport. However, the CD/g-C_3_N_4_(0.5) composite with the highest coverage of CDs still achieved the best bisphenol A (BPA) degradation rate at 3.87 times higher than that of g-C_3_N_4_. Hence, the charge separation efficiency should not be one of the main factors responsible for the enhancement of the photocatalytic activity of CD/g-C_3_N_4_. Instead, the light absorption capability was the dominant factor since the photoreactivity correlated well with the ultraviolet–visible diffuse reflectance spectra (UV–vis DRS) results. Although the CDs did not display upconversion photoluminescence (UCPL) properties, the π-conjugated CDs served as a photosensitizer (like organic dyes) to sensitize g-C_3_N_4_ and injected electrons to the conduction band (CB) of g-C_3_N_4_, resulting in the extended absorption spectrum from the visible to the near-infrared (NIR) region. This extended spectral absorption allows for the generation of more electrons for the enhancement of BPA degradation. It was determined that the reactive radical species responsible for the photocatalytic activity were the superoxide anion radical (O_2_^•−^) and holes (h^+^) after performing multiple scavenging tests.

## Introduction

Carbon dots (CDs) predominantly consist of amorphous carbon together with nanocrystalline regions of sp^2^-hybridized graphitic carbon [1]. CDs possess upconversion photoluminescence (UCPL) properties [2] and are able to harvest long wavelength light in the visible and near infrared (NIR) region [3–4], rendering them promising candidates as photosensitizers in photocatalysis. Nevertheless, the reported preparation method of CDs has met several limitations including limited spectral efficiency, low product yield and toxic chemicals usage [5]. Thus, the use of renewable bioprecursors such as orange juice [6], soy milk [7], orange waste peels [8], watermelon rinds [9], hair [10] and cow manure [11] to produce CDs has drawn the attention of researchers. Such green CDs are widely applied in bioimaging [6,12], sensing [13] and solar cells [14]. However, the obtained CDs showed superior water solubility, limiting their down-to-earth applicability for photocatalysis in aqueous solution. It is well-known that the organic semiconductor g-C_3_N_4_ possesses visible light harvesting properties due to its narrow band gap energy (≈2.7 eV) [15]. Thus g-C_3_N_4_ has emerged as a promising polymeric semiconductor for photocatalytic reduction of carbon dioxide (CO_2_) [16–20], hydrogen evolution [21–25], oxidation of NO [26–27], and degradation of pollutants [28–30]. However, the photocatalytic performance of bulk g-C_3_N_4_ remains unsatisfactory because of the fast recombination rate of electron pairs and narrower light absorption range over the entire solar spectrum. Turning g-C_3_N_4_ into a mesoporous nanorod structure [31] and the hydrogenation of g-C_3_N_4_ [32] could be an alternative to increase the light-harvesting ability and charge separation efficiency. Our group has reported the self-modification of g-C_3_N_4_ structures using alkaline [28] and acid treatment [29] to overcome the limitations of g-C_3_N_4_. Despite of the positive results, the self-modification consumes concentrated alkali and acid, which is harmful to our environment and health. It has been accepted that the coupling of nanocarbon materials with other semiconductors [21] could induce synergetic effects like photosensitization, electron mediator and acceptor and increasing adsorption [33–34]. Therefore, g-C_3_N_4_ could be combined with CDs to overcome the solubility problem of CDs and to boost the performance of g-C_3_N_4_. Besides, the CDs can also act as photosensitizers to harvest a wide spectrum of solar light to achieve efficient daylight-driven photocatalysis. Prasannan and Imae reported a simple and facile one-pot synthesis of fluorescent CDs from orange waste peels using the hydrothermal carbonization method. As prepared CDs were combined with zinc oxide (ZnO) to degrade naphthol blue–black azo dye under UV irradiation, and the superior photocatalytic activity was demonstrated [8]. Guo and co-workers used the electrochemical method to produce CDs from graphite rods. The deposition of CDs onto g-C_3_N_4_/ZnO heterojunctions enhanced the degradation of tetracycline by absorbing a wider spectrum of visible light and suppressing the recombination of electron–hole pairs [35]. A facile hydrothermal approach was adopted to synthesize CD/g-C_3_N_4_ using ascorbic acid as precursor to prepare CDs, showing higher hydrogen (H_2_) production than pure g-C_3_N_4_ under UV light irradiation [36]. A similar composite was also reported using 6-aminohexanoic acid [37] and rapeseed flower bee pollen [38] as precursors to produce CDs for the photocatalytic generation of H_2_ under visible light irradiation. In addition, Wang and co-workers used citric acid and urea as precursors to prepare N-doped CDs (NCDs). The NCD/g-C_3_N_4_ composite exhibited better degradation of indomethacin from the UV to NIR spectrum because of the superior electron transfer and extension of visible light absorption region after doping with N atoms [39]. Their group further enhanced the efficiency of CD/g-C_3_N_4_ through the incorporation of single-atom-dispersed silver. The optimum amount of Ag (3.0 wt %) and CDs (1.0 wt %) resulted in a 10-fold higher degradation rate of naproxen [40]. Much of the work on g-C_3_N_4_ has been reported for environmental and energy remediation [41–42]. The above-mentioned research works suggested that the enhancement of the photocatalytic performance was attributed to the dual functionality of CDs as electron trapper and photosensitizer. Although some works have been carried out in this field, several insights have yet to be explored to fill the gaps of previous works, including (i) the utilization of harmless solar energy as a resource to irradiate photocatalytic degradation of organic pollutant (since CDs could act as a photosensitizer over the entire solar spectrum) and (ii) acknowledgement that the most reported bioprecursor-derived CDs and g-C_3_N_4_ composite are limited to the photocatalytic generation of H_2_ (instead limited attempts have been performed on the removal of the organic pollutant since foremost discovery by Prasannan and Imae [8]).

Collectively, considering all of the aforementioned problems, it provokes the idea that the combination of g-C_3_N_4_ with CDs produced from sugarcane juice can be used for the photocatalytic degradation of endocrine disrupting chemicals (EDCs). Bisphenol A (BPA) was chosen as a model pollutant under natural sunlight irradiation. Herein, the structural and optical properties of samples were characterized by X-ray diffraction (XRD), Fourier transform infrared spectroscopy (FTIR), photoluminescence (PL), X-ray photoelectron spectroscopy (XPS), transmission electron microscopy (TEM), etc. The influence of the weight percentage of CDs and a scavenging experiment were carried out to illustrate the potential degradation mechanisms. Overall, the incorporation of CDs into g-C_3_N_4_ was efficient towards the removal of BPA, mainly because the CDs act as a photosensitizer to extend the light harvesting region.

## Results and Discussion

### Field emission scanning electron microscopy (FESEM) and transmission electron microscopy (TEM)

**Figure 1:**
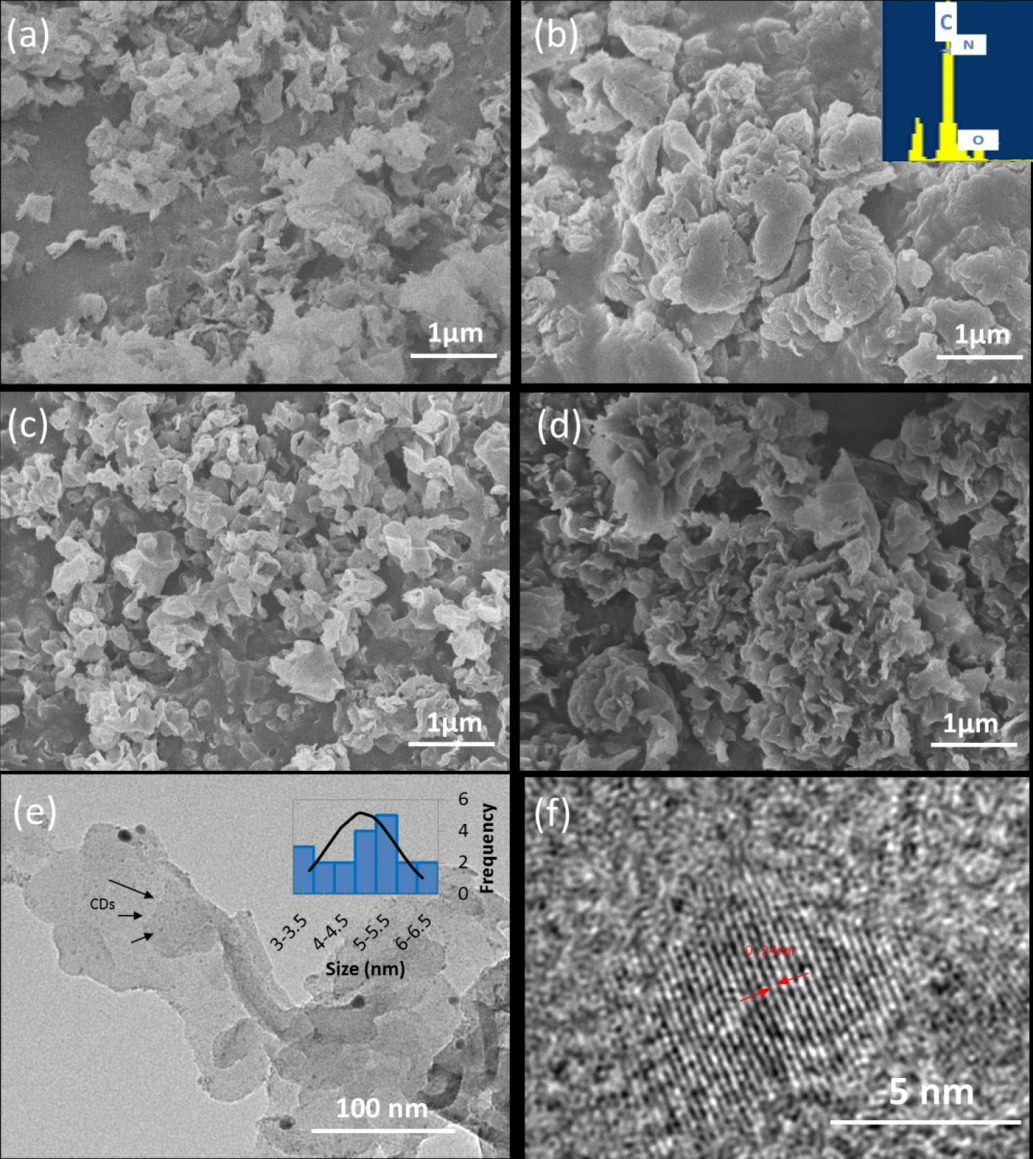
Field emission scanning electron microscopy (FESEM) images of (a) g-C_3_N_4_, (b) CD/g-C_3_N_4_(0.1), (c) CD/g-C_3_N_4_(0.2), and (d) CD/g-C_3_N_4_(0.5) composites. (e) TEM image and (f) HRTEM image of CD/g-C_3_N_4_(0.5). The insets of (b) and (e) show the energy-dispersive X-ray spectroscopy (EDS) results and particle size distribution of CDs, respectively.

Figure 1a shows a field emission scanning electron microscopy (FESEM) image of a lamellar structure of pure g-C_3_N_4_ with nonuniform, porous folding structures. The surface of the CD/g-C_3_N_4_ composites were conglomerate and resembled each other in various ways after incorporating different concentration of CDs as shown in Figure 1b–d. The wrinkled structure of g-C_3_N_4_ provides a great support to attach with CDs. After incorporating the CDs, the CD/g-C_3_N_4_ composites exhibited a more packed and agglomerated morphological nanostructure compared to that of pure g-C_3_N_4_. A similar observation was reported in our previous research [28], showing that the clustered effect was attributed to the self-assembly process during the hydrothermal treatment [43]. However, a more porous structure in the CD/g-C_3_N_4_(0.5) composite was observed as compared to that of CD/g-C_3_N_4_(0.1), signifying that the increasing concentration of CDs could reduce the agglomeration effect in the composites. The detection of elemental C and N (inset of Figure 1b) using energy-dispersive X-ray spectroscopy (EDS) was attributed to the presence of pristine g-C_3_N_4_ and CDs [44]. A small peak of elemental O inevitably appeared due to reaction under ambient conditions. Figure 1e and Figure S2 (Supporting Information File 1) show a TEM image of CD/g-C_3_N_4_(0.5) and g-C_3_N_4_, respectively. The observation of CDs (small dark spots) shows that the CDs were well distributed onto the smooth, thin-layered g-C_3_N_4_ nanosheets. The inset of Figure 1e indicates that the particle size of the CDs falls in the range of 3−6.5 nm. The lattice fringes of the CDs were found to be about 0.24 nm (Figure 1f), which correlated with the (100) in-plane lattice spacing of graphene [45].

### X-ray diffraction (XRD) and Fourier-transform infrared spectroscopy (FTIR)

**Figure 2:**
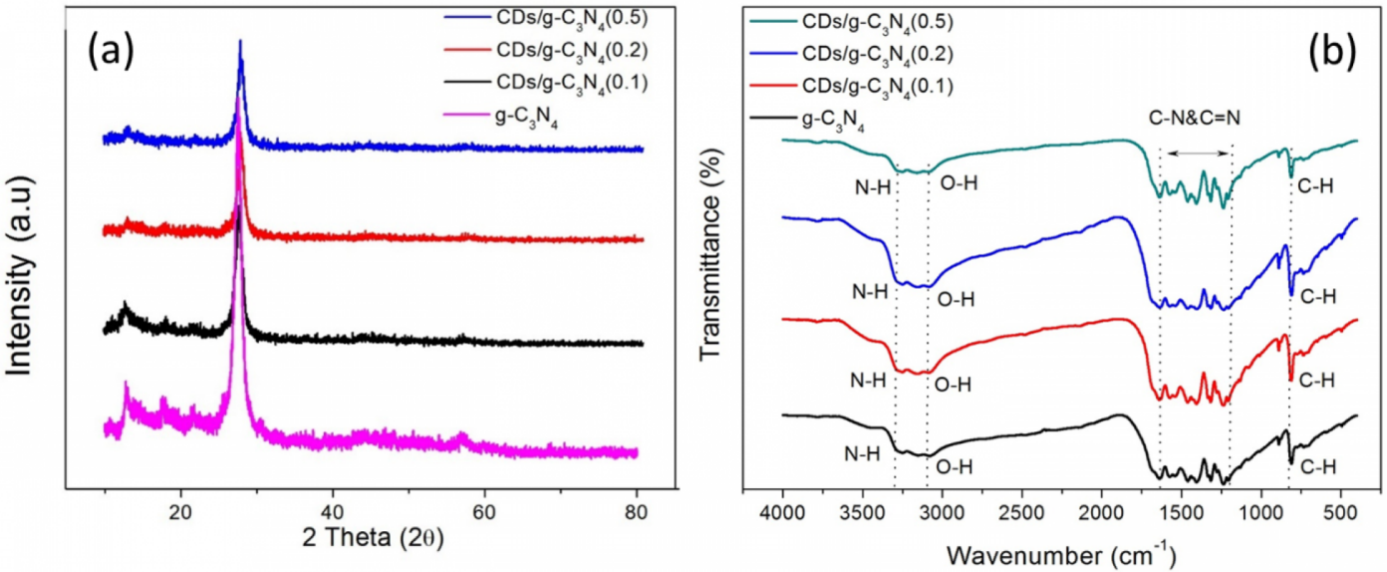
(a) XRD patterns and (b) FTIR spectra of g-C_3_N_4_, CD/g-C_3_N_4_(0.1), CD/g-C_3_N_4_(0.2), and CD/g-C_3_N_4_(0.5).

Figure 2a shows the XRD patterns of all CD/g-C_3_N_4_ composites. A major diffraction peak was found around 27°, representing the graphitic aromatic C–N units. The diffraction angle peak shifted from 27.5° to 27.8° after incorporating 0.2 and 0.5 wt % of CDs into the g-C_3_N_4_, indicating that the CDs were successfully intercalated into the host matrix and the defects were introduced in the g-C_3_N_4_ [46]. A low concentration of CDs at 0.1 wt % did not exert significant changes in the host structure. The strong peak at 27.6° was ascribed to the stacking of aromatic units with crystal face (002) with interplanar distance of 0.328 nm [47]. A minor peak at 12.8° was correlated to condensed tri-*s*-triazine units in the sheets (001) with an interplanar distance of 0.665 nm [48]. No extra peaks were detected in the CD/g-C_3_N_4_ composites due to the insignificant content of CDs [49–50]. In Figure 2b, the peaks at 3150 and 3155 cm^−1^ are attributed to the amide N–H stretch and carboxylic acid O–H stretch, respectively. The presence of N–H stretching vibration modes is due to some uncondensed amine functional groups in the carbon nitride layer [51]. The band in the range of 1230–1650 cm^−1^ corresponded to the stretching of sp^3^ C–N and sp^2^ C=N in CN heterocycles that exists within the g-C_3_N_4_ compound [52]. The sharp peak at 811 cm^−1^ corresponded to the alkyne C–H bond (trisubstituted) or the breathing mode of the heptazine arrangement [44]. The alkene C=C bond (conjugated) at 1639 cm^−1^ was due to the connection between heptazine-based g-C_3_N_4_ [53]. As shown in Figure S1 (Supporting Information File 1), the CDs show the obvious absorption peaks at 2925 cm^−1^, 1608 cm^−1^ and 670 cm^−1^ which are correlated to the stretching vibrations of C–H, stretching vibrations of C=O, and bending vibration of =C–H, respectively. The three obvious absorption peaks at 2368 cm^−1^, 1409 cm^−1^ and 1095 cm^−1^ are associated with the stretching and bending vibrations of C≡N, C=C and C−O−C, respectively. This result is consistent with previous reports [54–55]. Similar absorption bands were observed for all CD/g-C_3_N_4_ composites because the strong peaks from g-C_3_N_4_ covered the weak absorptions of CDs due to the low CD content.

### Optical properties

**Figure 3:**
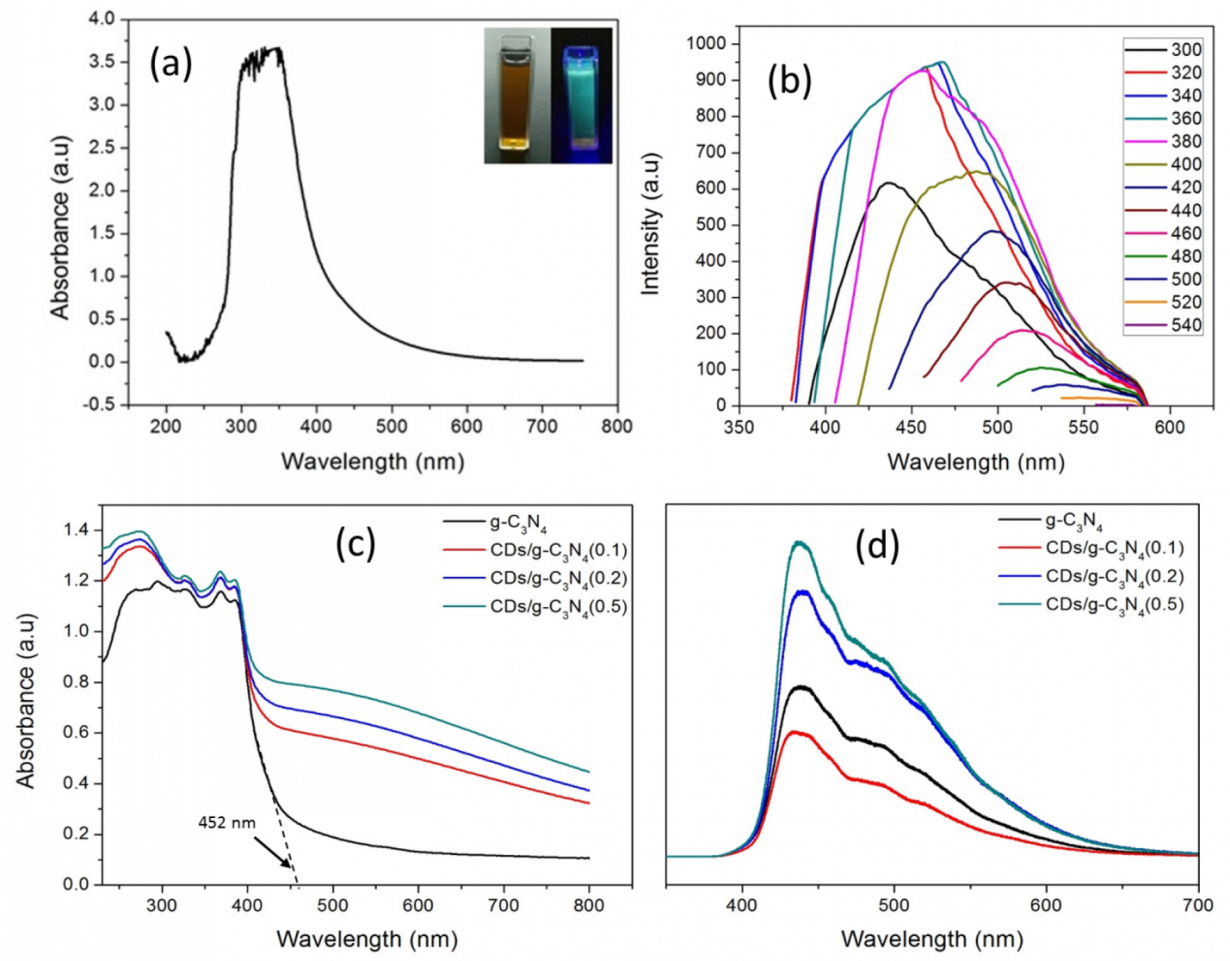
(a) The absorption spectrum of carbon dots (CDs) solution. The inset shows the fluorescence of the CDs under daylight (left) and UV light irradiation at 365 nm (right). (b) The photoluminescence (PL) emission spectrum of CDs at different excitation wavelengths, (c) UV–vis diffuse reflection spectra (DRS) spectra, and (d) PL spectra of the composites.

Figure 3a shows the UV–vis absorption spectra of CDs in an aqueous solution in the range from 200 nm to 750 nm. The obvious peak at about 330 nm could be ascribed to the n–π* transition of C=O bond [56]. The inset of Figure 3a shows that the CD solution emitted a strong blue-green fluorescence under excitation at 365 nm with a UV lamp. The CD solution was tested with different excitation wavelengths to evaluate the ideal excitation spectrum that initiates the photoluminescence (PL) properties. Figure 3b displays wide, broad PL emission peak across the UV to visible spectrum ranging from 380 to 580 nm. It was found that the optimal excitation and emission wavelengths are ≈320−380 nm and ≈460 nm, respectively. The emission spectrum of the CD solution was red-shifted to longer wavelengths with decreasing intensity when the excitation wavelength increased, which is consistent with previous reports [4,56]. Although the origin of the PL luminescence in CDs is not fully elucidated, the excitation-dependent PL behavior of CDs is usually correlated to the size distribution of CDs, the recombination of photogenerated charges at surface-confined defect states and the different distributions of emissive trap sites [57–59]. The UCPL of CDs only occurs when multiphotons are activated simultaneously, emitting emission wavelengths shorter than the excitation wavelength [60]. In Figure 3b, no emission peak was detected when the excitation wavelength was above 540 nm, which is consistent with previous reports [61]. A recent study by Wen and co-workers suggested that most of the CDs and graphene quantum dots (GQDs) do not have detectable upconversion fluorescence. The frequently cited UCPL properties could originate from the normal fluorescence excited by the leaking component from the second diffraction in the monochromater of the fluorescence spectrophotometer [62].

As shown in Figure 3c, the pure g-C_3_N_4_ showed expected absorption peaks at 265 nm and 340 nm due to the π–π* or n–π* electronic transitions in the conjugated aromatic s-heptazine ring system of g-C_3_N_4_ [63]. The onset of the absorption spectra of pure g-C_3_N_4_ started from 452 nm, showing a band gap of 2.75 eV, which is almost the same as that previously reported [28,46,64]. It is obvious that all CD/g-C_3_N_4_ composites displayed a broad light absorption in the whole solar spectrum ranging from 200 to 800 nm due to the quantum confinement effect induced by the CDs [65]. This result suggests that CD/g-C_3_N_4_ composites possess greater potential than that of pure g-C_3_N_4_ to drive photocatalysis under the irradiation of the entire solar spectrum. In contrary to our earlier work that reported blue-shifted absorption spectra after titanium dioxide (TiO_2_) loading [28] and acid treatment [29], the incorporation of CDs into g-C_3_N_4_ consistently red-shifted the absorption spectra towards the visible and NIR region. However, the photocatalytic performance of the composites is possibly limited under NIR light irradiation because the UCPL properties of CDs (which could overcome the low energy of NIR-light photons to initiate the excitation of electrons) are not detected in Figure 3b. The PL spectra in Figure 3d were evaluated to determine the charge separation efficiency of all composites. The recombination of electron–hole pairs produces emission of photons resulting in photoluminescence. The weaker PL intensity corresponded to a lower recombination rate of excited electron [66]. Among the composites, CD/g-C_3_N_4_(0.1) (with a lower CD coverage) exhibited the lowest PL intensity, indicating that the minimum coverage of CDs could suppress the recombination of photogenerated carriers in g-C_3_N_4_. As the CD coverage increased, trap states were created at the CD/g-C_3_N_4_ interface, thus increasing the trapping events and decelerating the electron transport [67–69]. A similar observation was reported previously and it was concluded that CDs are favorable for the separation of charge carriers, but the increasing concentration of CDs could lead to inefficiency of charge separation [46].

### X-ray photoelectron spectroscopy (XPS) analysis

**Figure 4:**
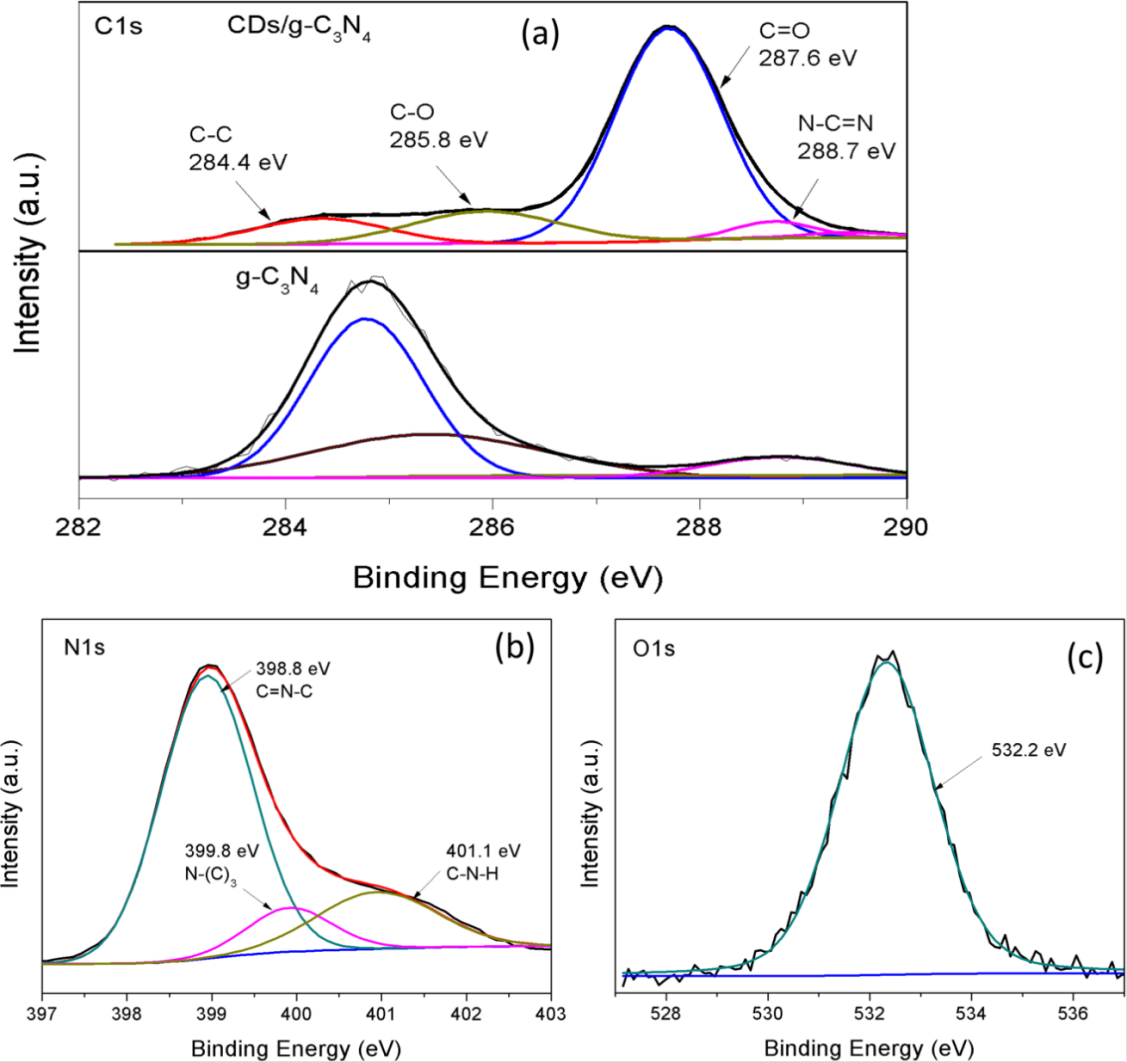
X-rat photoelectron spectroscopy (XPS) of (a) C 1s (b) N 1s, (c) O 1s for CD/g-C_3_N_4_(0.5), and (d) C 1s for g-C_3_N_4_.

The XPS spectra in Figure 4a–c reveal the surface composition and chemical state of CD/g-C_3_N_4_(0.5). From the C 1s spectrum of CD/g-C_3_N_4_(0.5) in Figure 4a, the signals at 284.4 eV and 288.7 eV are found to correspond to graphitic carbon (C**–**C) and sp^2^ carbon (N**–**C=N) [70]. Compared with the C 1s spectrum of pure g-C_3_N_4_, three new signals were detected at 284.8 eV (sp^2^ C**–**C), 285.4 eV (C–N) and 288.8 eV (N**–**C=N) after the incorporation of CDs, revealing the abundant carbonyl and carboxyl groups on the CDs as a result of the significant interaction between g-C_3_N_4_ and CDs [71–74]. The N 1s spectrum was deconvoluted into three peaks at 398.8 eV, 399.8 eV and 401.1 eV. The strong peak at 398.8 eV corresponds to sp^2^ hybridized aromatic N in the triazine units (C=N**–**C) while the signals at 399.8 eV and 401.1 eV are attributed to tertiary (N**–** (C)_3_) groups and amino functional groups (C**–**N**–**H) [75]. The sole O 1s binding energy at 532.2 eV is due to absorbed water [37].

### Photocatalytic performance under solar irradiation

**Figure 5:**
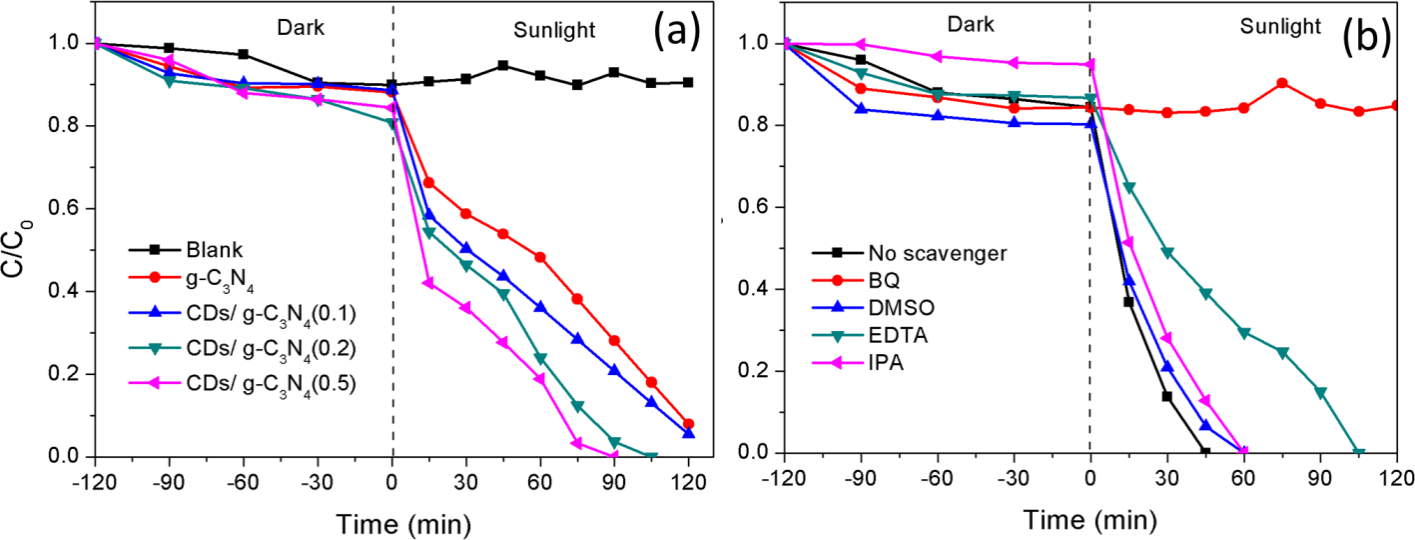
(a) Photocatalytic degradation of bisphenol A (BPA) as a function time under irradiation of natural sunlight, and (b) the effect of different scavengers for CD/g-C_3_N_4_(0.5) on BPA removal.

The photocatalytic performance of the composites was evaluated using BPA as a model pollutant under natural sunlight irradiation. Figure 5a shows that the blank was stable with almost no degradation throughout the experiment, indicating BPA had poor photolysis properties. The CD/g-C_3_N_4_(0.5) exhibited the best photocatalytic degradation rate with 100% removal of BPA after 90 min of reaction. Pure g-C_3_N_4_ achieved relatively good degradation efficiency (68.2%) attributed to its visible light absorption capability (Table S1, Supporting Information File 1). A great difference in the degradation rate was observed between CD/g-C_3_N_4_(0.1) and CD/g-C_3_N_4_(0.2) in which the degradation efficiency increased to ≈20% with the addition of 0.1 wt % of CDs. Both CD/g-C_3_N_4_(0.1) and g-C_3_N_4_ were unable to fully eliminate BPA within the 2 h of reaction time. These results correlated well with the UV DRS results (Figure 3c), in which the increasing concentration of CDs resulted in stronger photosensitizing effect in the composite, thus leading to a broader absorption band in the visible and NIR light region compared to that of pure g-C_3_N_4._ This indicates that the electrons can be easily excited in each of the CDs and g-C_3_N_4_ to produce more electron–hole pairs for the reaction of pollutants. The recombination rate of electron–hole pairs was another factor affecting the degradation rate. As shown in the PL analysis (Figure 3d), the separation efficiency of electron–hole pairs will be suppressed if the concentration of CDs is too high. However, the photocatalytic performance did not correlate well with the PL analysis. The remarkable performance of CD/g-C_3_N_4_(0.5) proved that the intense photosensitizing effect of CDs at higher dosage could overcome the poorer separation efficiency of charge carriers, leading to the enhancement of photocatalytic activity. As a result, the photocatalytic performance followed the sequence of CD/g-C_3_N_4_(0.5) > CD/g-C_3_N_4_(0.2) > CD/g-C_3_N_4_(0.1) > g-C_3_N_4_ > blank.

**Figure 6:**
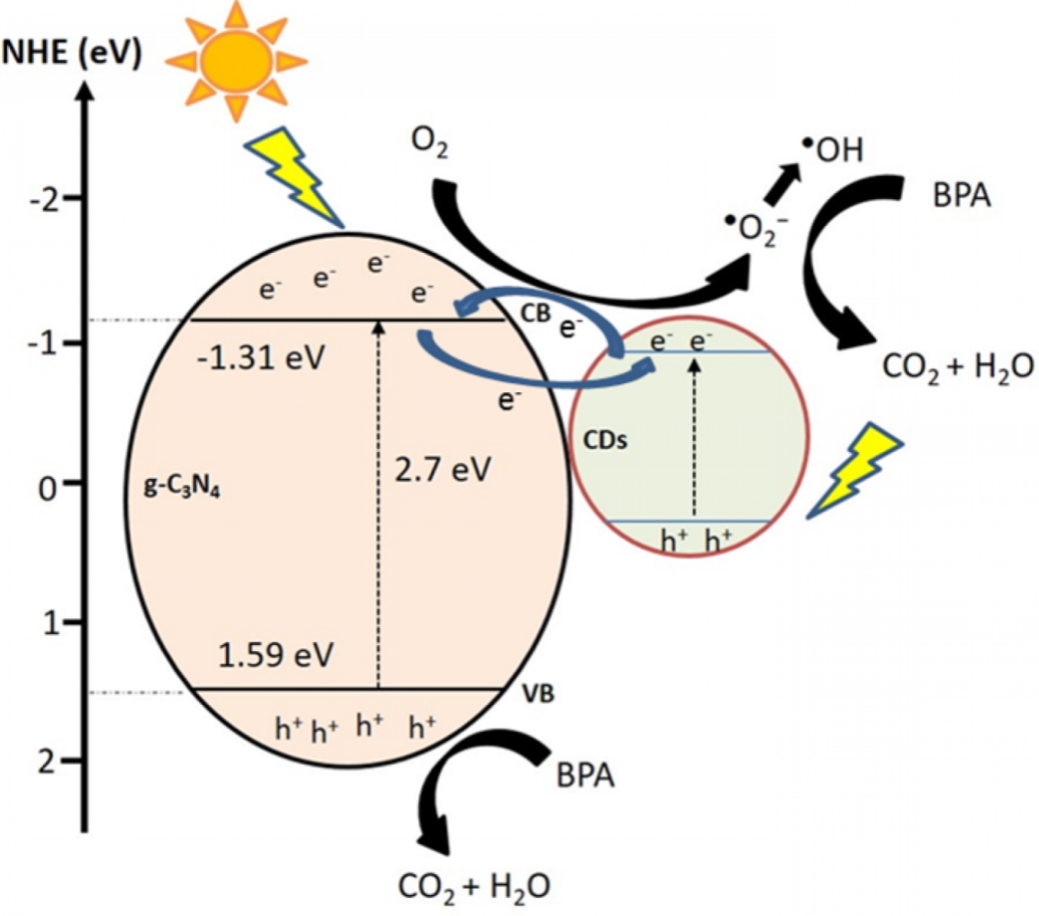
Electron transfer mechanism of CD/g-C_3_N_4_.

A radical scavenging experiment (Figure 5b) was carried out to identify the main radicals involved in the photoreaction. The influence order of the active radical species on the degradation of BPA was O_2_^•−^ > h^+^ > hydroxyl radical (^•^OH) > e^−^. It is obvious that O_2_^•−^ was the main radical species generated since the photodegradation of BPA was significantly inhibited after the addition of benzoquinone (BQ). The addition of ethylenediaminetetraacetic acid disodium salt dihydrate (EDTA-2Na^+^) in BPA solution slightly suppressed the degradation rate, showing that h^+^ was also one of the active radicals involved in the photoreaction. In contrast, neither ^•^OH nor e^−^ are the main reactive species because the addition of dimethylsulfoxide (DMSO) and isopropyl alchohol (IPA) did not exert a significant effect on the degradation rate of BPA. Based on the above results, a degradation mechanism is proposed in Figure 6.

The edge potential of the conduction band (CB) and the valence band (VB) of a semiconductor at the point of zero charge was estimated using the following equations:

[1]
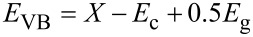


[2]
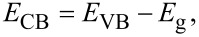


where *E*_VB_ and *E*_CB_ are the VB and CB edge potential respectively, *X* is the electronegativity of the semiconductor; *E*_c_ is the energy of free electrons on the hydrogen scale (≈4.5 eV vs NHE) and *E*_g_ is the band gap energy of the semiconductor. The *X* value of g-C_3_N_4_ is 4.64 eV [75] while the band gap energy of g-C_3_N_4_ is 2.75 eV. The VB and CB of g-C_3_N_4_ were theoretically calculated at 1.52 eV and −1.23 eV, respectively. In g-C_3_N_4_, the electrons were easily excited from the VB to the CB because of its narrow band gap energy. The trapped h^+^ in the VB directly oxidized BPA but cannot react with water (H_2_O) and hydroxide (OH^−^) to generate ^•^OH because the VB of g-C_3_N_4_ (+1.52 eV vs NHE) was more negative than the redox potential of OH^−^/^•^OH (+2.4 eV) [76]. With a lower amount of CDs, the CDs facilitated efficient electron transfer from the CB of g-C_3_N_4_ to adsorbed oxygen (O_2_), generating O_2_^•^. Meanwhile, O_2_^•−^ radicals also reacted with the hydrogen (H^+^) ion to further produce a minor portion of ^•^OH radicals. Thus, the O_2_^•−^ and ^•^OH could oxidize the BPA simultaneously to enhance the photocatalytic performance. Although the CDs did not display UCPL properties, the π-conjugated CDs served as a photosensitizer (like organic dyes) to sensitize g-C_3_N_4_ and injected electrons to the CB of g-C_3_N_4_ [37–38,73], resulting in an extended absorption spectrum from the visible to the NIR region. This results in the generation of more electrons for the enhancement of BPA degradation. It is notable that the photosensitizing effect of CDs serves as a dominant factor for enhancing the photocatalytic activity of CD/g-C_3_N_4_ composites since the degradation rate of BPA is well consistent with the UV DRS results. Unlike CDs, TiO_2_ in combination with g-C_3_N_4_ for the photocatalytic process [28] was able to trap electrons efficiently due to the favorable band edge potential; however, the material could not absorb visible light. As shown in Table S2 (Supporting Information File 1), CD/g-C_3_N_4_ composites demonstrated the best degradation efficiency of BPA than that of other composites reported earlier in our previous works [28–29].

## Conclusion

The CD/g-C_3_N_4_ composite photocatalyst was successfully synthesized using hydrothermal treatment. With an increase in the concentration of CDs, the UV–vis DRS results showed a broader and stronger visible and NIR light absorption band while the lattice distortion of g-C_3_N_4_ was observed in XRD analysis. The minimal coverage of CDs (<0.5 wt %) mediated the electron transfer from the CB of g-C_3_N_4_ to adsorbed O_2_ to produce O_2_^•−^. It is evidenced by the scavenger test that both O_2_^•−^ and h^+^ were the major active species involved in the redox process. The excessive loading of CDs could act as recombination centers to increase the recombination rate of electron–hole pairs, but this did not reduce the photocatalytic performance of CD/g-C_3_N_4_(0.5). In fact, the degradation rate of BPA in all CD/g-C_3_N_4_ composites increased with the increasing concentration of CDs, which correlated well with UV–vis DRS results. The higher coverage of CDs exerted a stronger photosensitizing effect in CD/g-C_3_N_4_(0.5) to overcome its inefficient charge carrier separation, improving the BPA removal efficiency by 3.87-fold as compared to g-C_3_N_4_. The preparation of CD/g-C_3_N_4_ is considered cost-effective and environmentally friendly because sugarcane juice was used as a “green” precursor instead of harmful chemicals. The photocatalytic reaction occurred under natural sunlight irradiation, which is renewable and sustainable.

## Experimental

### Materials and reagents

All the chemical reagents used were of analytical grade and purity. Sugarcane juice was obtained from a night market where the deposit was filtered out. The dichloromethane (≥99.8%), BPA (>99%) and BQ (>98%) were purchased from Sigma-Aldrich. The ethanol (95%) and urea (>99.5%) were purchased from R&M Chemicals. Distilled water was used throughout the experiment for dilution and washing purpose. In addition, IPA (83.5%) and DMSO (99%) was purchased from Bendosen and Univar, respectively. The EDTA-2Na^+^ (99%) was obtained from ChemSoln.

### Preparation of carbon nitride (g-C_3_N_4_) and carbon dot solutions

Urea was chosen as a starting material to produce g-C_3_N_4_. It was placed into an alumina crucible with a cover to avoid overreaction with oxygen. The urea was heated at 550 °C for 3 h to obtain yellowish g-C_3_N_4_. The synthesis of CDs was similar to the method reported by Sahu and co-workers [6]. To prepare the CD solution, 40 mL of sugarcane juice was mixed with 30 mL ethanol and stirred for 10 min to ensure homogeneity. The mixture was then transferred to a 80 mL teflon-lined stainless-steel autoclave and closed up tightly. It was dried in oven at 120 °C for 8 h with a heating rate of 1 °C/min. A dark brown solution was obtained and it was washed with dichloromethane to remove unreacted organics moieties. The aqueous solution was centrifuged at 3000 rpm for 15 min to separate out the residue and less fluorescent deposit. Two layers of solution formed where the top layer was extracted into a beaker. The acetone solution was added excessively into the solution and centrifuged at 10,000 rpm for 15 min to remove the remaining deposit. A clear yellowish-brown solution was obtained.

### Preparation of CD/g-C_3_N_4_ composites

0.2 g of g-C_3_N_4_ was mixed with the previously prepared CD solution and then stirred for 24 h using a hot plate magnetic stirrer under room temperature. After the solution mixed homogeneously, it was placed into teflon-lined stainless-steel autoclave and heated at 100 °C for 2 h. It was then centrifuged to remove excessive CDs. The samples were then dried overnight. The synthesis route is illustrated in Figure S3 (Supporting Information File 1). Same preparation steps were repeated to synthesize the composites with 0.1, 0.2 and 0.5 wt % of CDs. The samples obtained were denoted as CD/g-C_3_N_4_(0.1), CD/g-C_3_N_4_(0.2) and CD/g-C_3_N_4_(0.5), respectively.

### Characterization of CD/g-C_3_N_4_ composites

FTIR spectra (Perkin Elmer Spectrum 400 spectrophotometer) were conducted in the range of 400–4000 cm^−1^ with the samples dispersed in KBr. The XRD analyses were performed using the powder XRD (PANalytical-Empyrean) with Cu Kα radiation at a scanning speed of 0.02 s^−1^. A micro-PL spectroscope (Renishaw, inVia Raman Microscope) was used to acquire the PL spectra with an excitation wavelength of 325 nm. UV–vis DRS was obtained using a Shimadzu UV-2600 spectrophotometer equipped with an integrating sphere attachment with barium sulfate (BaSO_4_) as a reference. The surface chemical composition of samples was analyzed by XPS (PHI Quantera II, Ulvac-PHI, Inc.) with an Al Kα radiation source. High resolution transmission electron microscope (HRTEM, FEI-TECNAI F20) images were obtained at 200 kV. PL spectra of CDs solution were acquired with a PL spectrophotometer (Perkin Elmer LS 55) with different excitation wavelengths ranging from 300 to 540 nm.

### Photocatalytic degradation of BPA

The photocatalytic performance of CD/g-C_3_N_4_ composites was investigated using BPA under natural sunlight irradiation. All photocatalytic experiments were carried out under sunlight radiation for a duration of 2 h. The initial concentration of BPA was 5 mg/L prepared in a 500 mL beaker with 250 mL solution. Each experiment was carried out for 2 h where a sample was collected at 15 min intervals. Each beaker was stirred well with 0.1 g of photocatalyst. Prior to the photocatalytic degradation, the solutions were magnetically stirred in the dark for 2 h to establish an adsorption–desorption equilibrium. The residual concentration of BPA was analyzed with a high performance liquid chromatography (HPLC, Flexar, Perkin Elmer) equipped with a UV detector (217 nm). An RS pak DE-413L (250 mm × 4.6 mm i.d., Showa Denko Co.) separation column was used. The mobile phase was 40% acetonitrile (ACN), 60% water and the flow rate was 0.7 mL min^−1^. The experiment was carried out under clear sky conditions at the University Tunku Abdul Rahman, Kampar (latitude 101.1398 °E and longitude 4.3394 °N) between 11:00 a.m. and 1:00 p.m. in May 2017. The solar light intensity was measured using a LT Lutron LX-101 lux meter of 1000 × 100 and the average light intensity over the duration of the clear sky weather conditions was found to be 89,200 lux. Table S3 (Supporting Information File 1) showed the recorded light intensity during the photocatalytic experiment.

### Scavenging experiment

CD/g-C_3_N_4_(0.5) was used as a photocatalyst in the scavenging experiment because of its superior photocatalytic performance among the samples. The scavenger test was used to identify the mechanism of photocatalyst to investigate the involved free radicals. Scavengers like BQ, DMSO, EDTA-2Na^+^ and IPA were used to conduct this test. BQ was used to trap O_2_^•−^ while DMSO functioned as the photoinduced e^−^ catcher. EDTA-2Na^+^ caught the h^+^ left by the excited electron. IPA worked to trap ^•^OH that was produced by the CD/g-C_3_N_4_ composites [77]. The scavenging test was carried out for 2 h under the solar radiation and the sample was collected for HPLC analysis.

## Supporting Information

File 1 Additional experimental data.
