# Supplementary material for: Sugarcane juice derived carbon dot–graphitic carbon nitride composites for bisphenol A degradation under sunlight irradiation
Source: Beilstein J Nanotechnol. 2018 Jan 30;9:353–63. doi: 10.3762/bjnano.9.35 (PMC5815291; doi:10.3762/bjnano.9.35)
Supplement: File 1 — Additional experimental data. [file Beilstein_J_Nanotechnol-09-353-s001.pdf]

Sugarcane juice derived carbon dot–graphitic carbon nitride composites for bisphenol A degradation under sunlight irradiation

Lan Ching Sim*[1], Jing Lin Wong[1], Chen Hong Hak[1], Jun Yan Tai[1], Kah Hon Leong[1], and Pichiah Saravanan[2]

Address: [1]Department of Environmental Engineering, Faculty of Engineering and Green Technology, Universiti Tunku Abdul Rahman, Jalan Universiti, Bandar Barat, 31900, Kampar, Perak, Malaysia and [2]Environmental Nanotechnology Laboratory, Department of Environmental Science and Engineering, Indian Institute of Technology (ISM), Dhanbad 826004, Jharkhand, India

Email: Lan Ching Sim - simcl@utar.edu.my

* Corresponding author

Additional experimental data

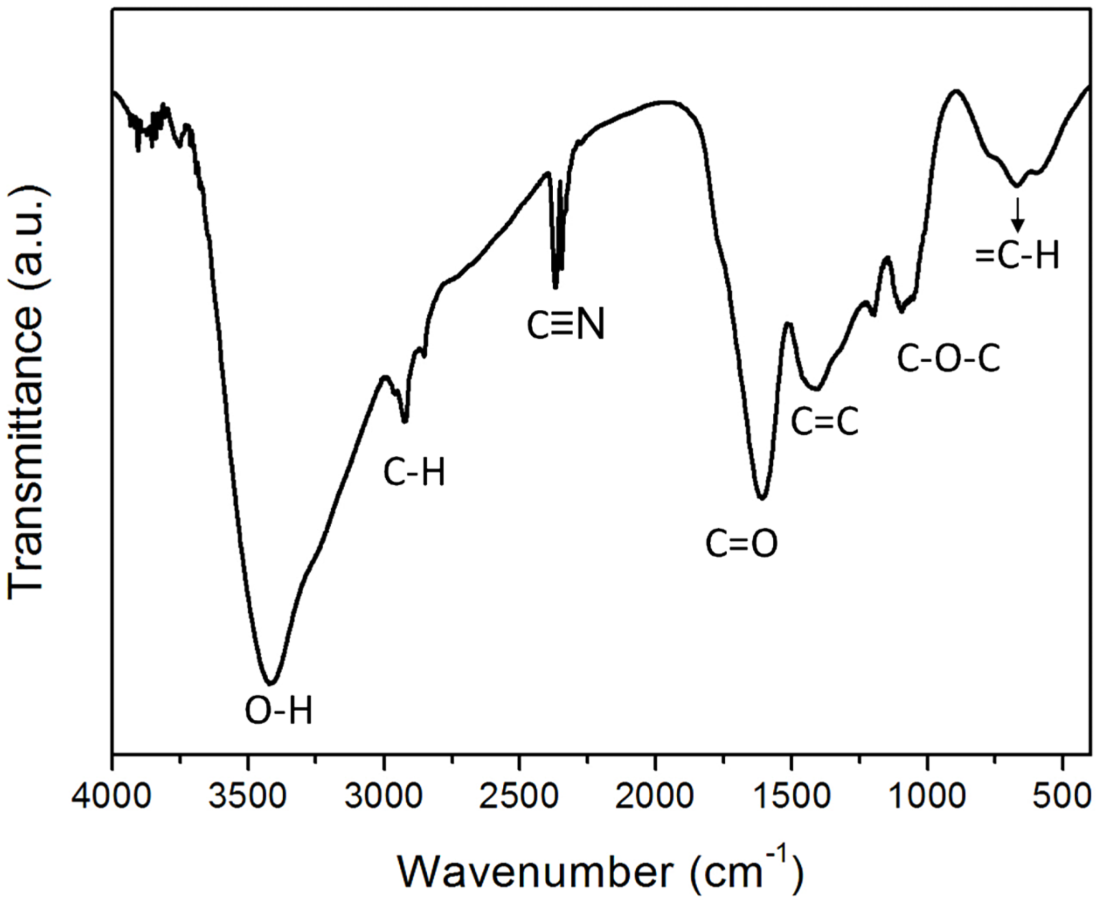

Figure S1: FTIR spectrum of CDs.

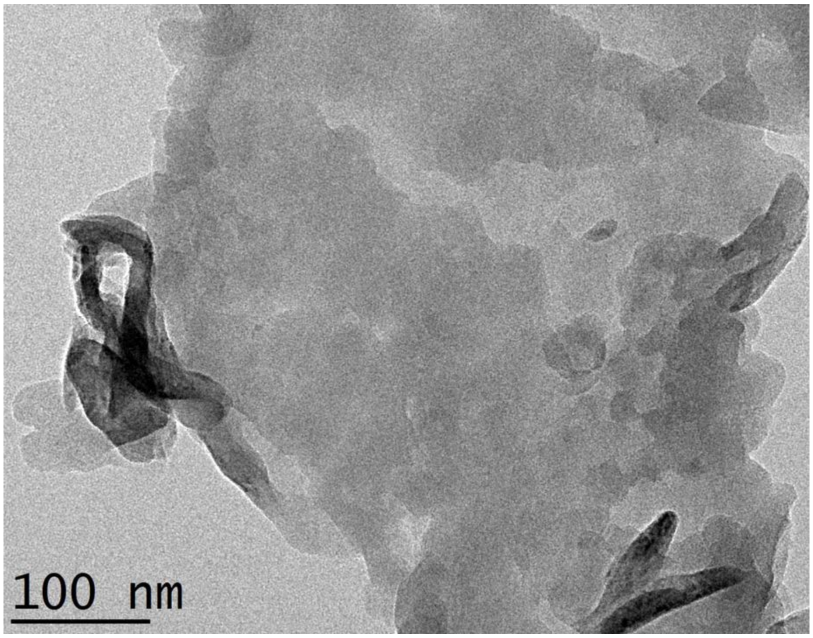

Figure S2: TEM image of g-C_3N_4.

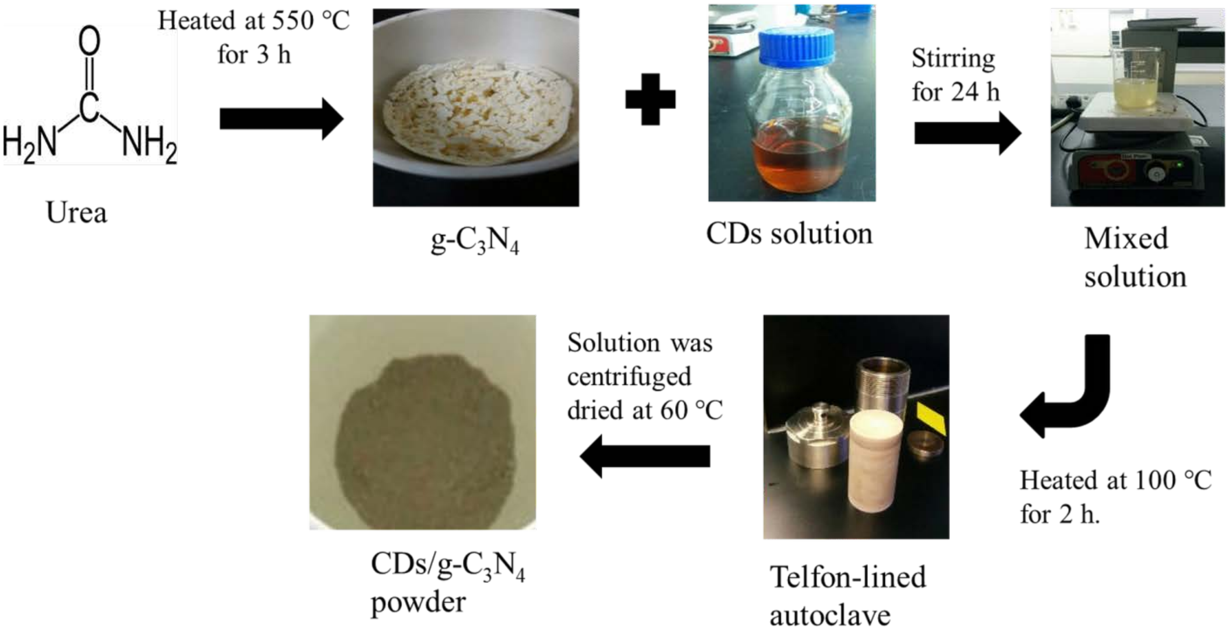

Figure S3: Synthesis route of CDs/g-C_3N_4 composites.

Table S1: The photodegradation rate of BPA by synthesized photocatalysts.

Sample	Photodegradation rate (%)	K (min^{-1})
g-C_3N_4	68.20	0.01118
CDs/g-C_3N_4(0.1)	76.66	0.01521
CDs/g-C_3N_4(0.2)	95.51	0.02501
CDs/g-C_3N_4(0.5)	100	0.04329

Table S2: Comparison of the catalytic efficiency between the nanocomposites synthesized in this work and previous works see [15,16] in main text.

**Sample**	**BPA Degradation Rate (%)**	**Reaction Time (min)**	**Ref. (see main text)**
CDs/g-C_3N_4(0.1)	76.7	90	*
CDs/g-C_3N_4(0.2)	95.5	90	*
CDs/g-C_3N_4(0.5)	100	90	*
HT-g-C_3N_4/TiO_2	46.9	360	[15]
g-C_3N_4/TiO_2	45.8	360	[15]
g-C_3N_4	55	210	[16]
acid treated g-C_3N_4	100	210	[16]

* current work

Table S3: The sunlight intensity for photodegradation experiments.

Time Interval (min)	0	15	30	45	60	75	90	105	120
Light intensity (100 lux)	823	867	904	920	870	862	934	950	900
